# Exposure of both the radius and ulna through a single posterior incision: a technical note

**DOI:** 10.1051/sicotj/2015024

**Published:** 2015-07-23

**Authors:** Abdel-Azim Hassan Wahsh

**Affiliations:** 1 National Institute for Neuromotor System 12652 Giza, Cairo Egypt

**Keywords:** Exposure, Radius and ulna, Single posterior incision

## Abstract

Fractures of both the radius and ulna are usually treated with two separate incisions and rarely with one single incision. However, both methods have disadvantages. For this we describe a relatively safe single straight posterior incision for exposure of the whole shafts of both the radius and ulna with the forearm rested on a board across the chest. This procedure was used in 116 forearms in 115 patients. The incision was in a straight line from the lateral humeral epicondyle to the ulnar head. The ulna was exposed between the extensor carpi ulnaris muscle and flexor digitorum profundus muscle covered by the aponeurosis of the flexor carpi ulnaris muscle and the radius between the extensor digitorum muscle and the extensor carpi radialis brevis muscle. During operation there was no difficulty in reducing or fixing any of the fractures in the whole shafts of the radius and ulna and at follow-up (average 5.2 years) there was no radioulnar synostosis or neurovascular injury in any of the forearms.

## Introduction

Fractures of both the radius and ulna are common. Usually open reduction and internal fixation of such fractures are done through two separate incisions with the forearm rested on a side board. There are three published approaches describing exposure of both the radius and ulna through a single posterior incision which are the Boyd approach [1], the combined approach described by Colton and Hall [2], and the biplanar approach described by Shenoy [3]. Both the approach using two separate incisions and the single approaches have disadvantages. The aim of our technique was to expose both the radius and ulna through a single straight posterior incision with the forearm rested on a board across the chest.

## Surgical technique

**Figure 1.**
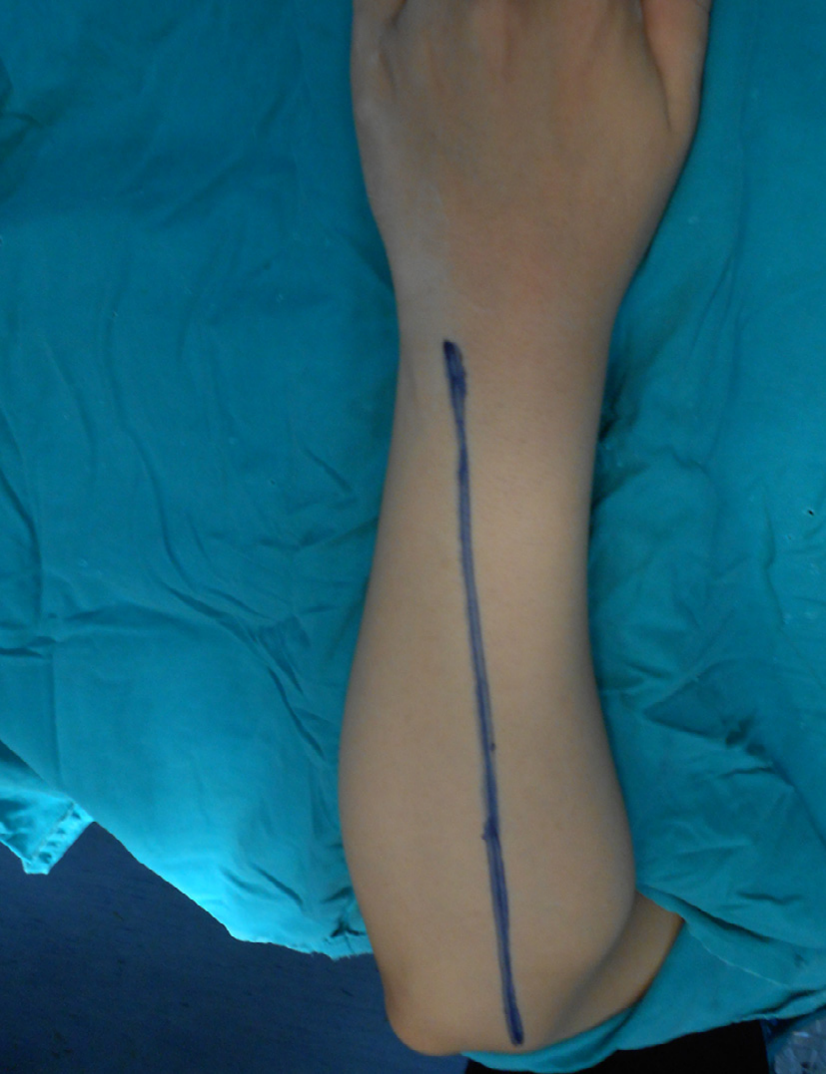
An intraoperative photograph demonstrating the incision in a straight line extending from the lateral humeral epicondyle to the ulnar head.

**Figure 2.**
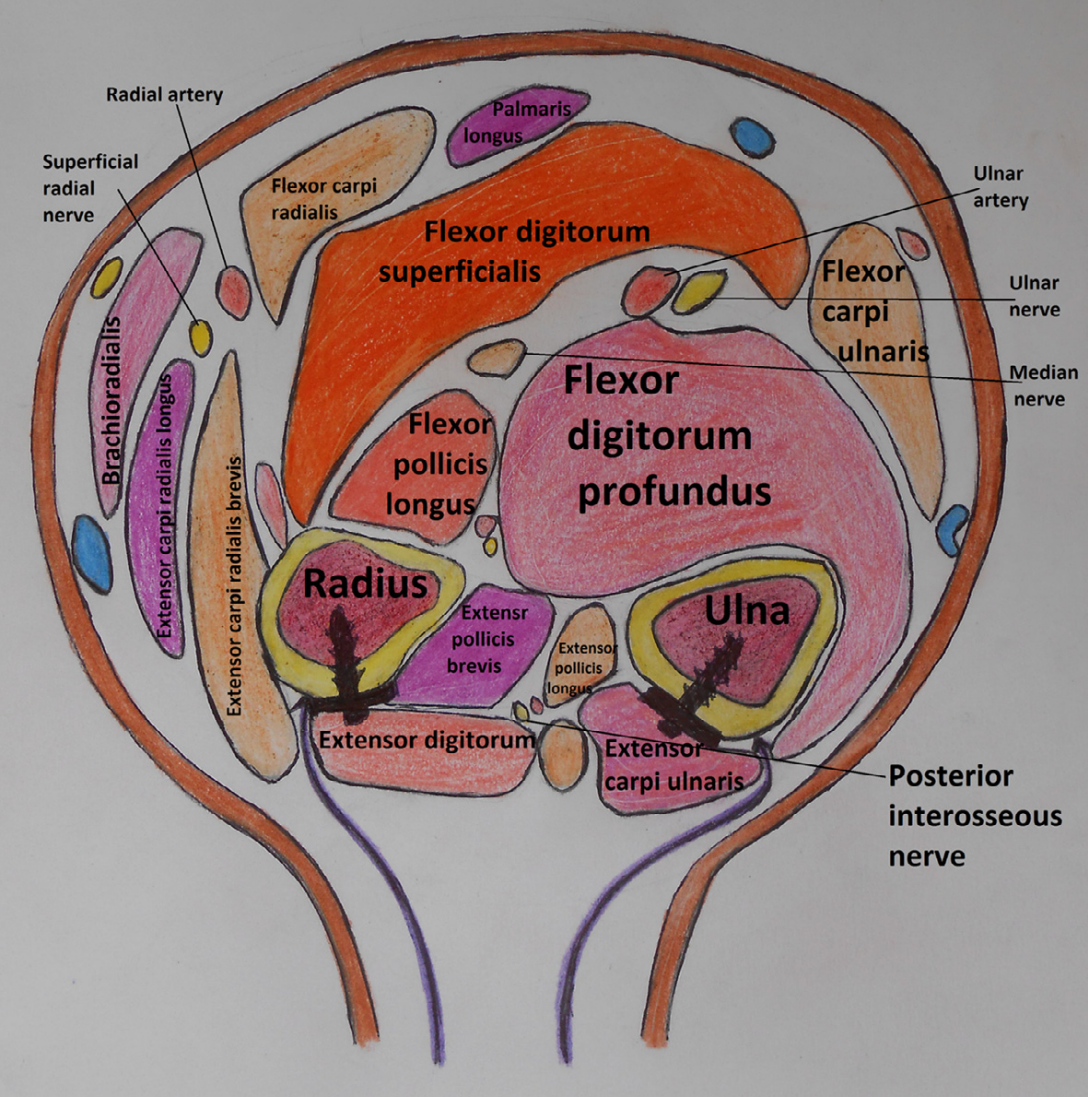
A cross-section in the middle of the forearm demonstrating the exposure of both the radius and the ulna through the single incision and showing the actual exposure of the ulna between the extensor carpi ulnaris and the flexor digitorum profundus muscles. It also demonstrates the relation of the exposure to the neurovascular structures and the position of the plates on the bones.

**Figure 3.**
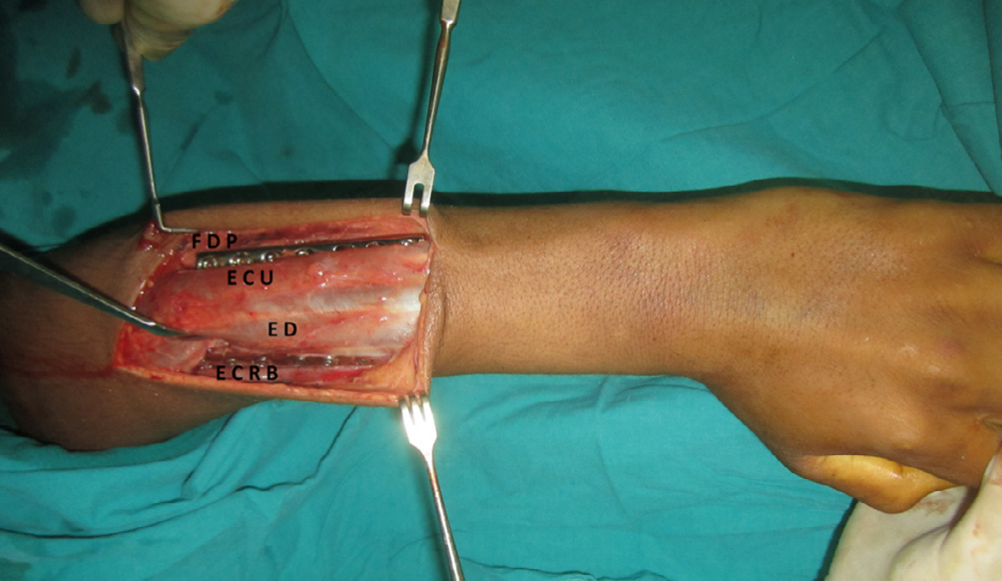
An intraoperative photograph demonstrating exposure of the radius between the extensor digitorum muscle (ED) and the extensor carpi radialis brevis muscle (ECRB), and the ulna between the extensor carpi ulnaris muscle (ECU) and the flexor digitorum profundus muscle (FDP) and fixation of each bone by a plate and screws.

**Figure 4.**
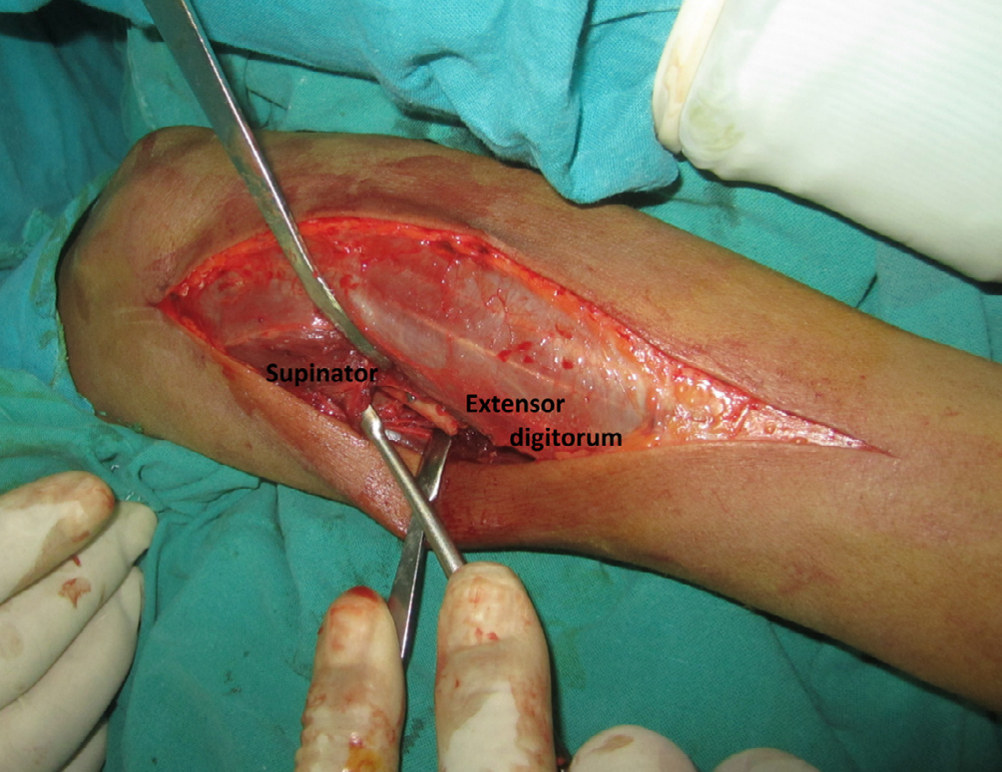
An intraoperative photograph demonstrating subperiosteal retraction of the supinator muscle.

The patient was laid supine with the forearm rested on a board across the chest. The incision was in a straight line from the lateral humeral epicondyle to the ulnar head (Figure 1). The length and site of the incision varied according to the level of the fractures. The skin and subcutaneous tissues were retracted exposing the deep fascia which is incised over the site of exposure of each bone separately. The ulna was exposed between the extensor carpi ulnaris muscle supplied by the posterior interosseous nerve and the flexor digitorum profundus muscle supplied by the ulnar nerve in its medial half and covered by the aponeurosis of the flexor carpi ulnaris muscle. The radius was exposed between the extensor carpi radialis brevis muscle and the extensor digitorum muscle (Figures 2 and 3). Both muscles were supplied by the posterior interosseous nerve. In the proximal third of the radius, the supinator muscle was elevated and retracted subperiosteally using the small fragment periosteal elevator and avoiding any injury to its substance (Figure 4). In the distal third, the abductor pollicis longus and extensor pollicis brevis muscles were gently retracted from the radius. After exposure of both the radius and ulna, reduction of the fractures was done and observed for both of them at the same time, then internal fixation was done. The plates were fixed on the tension side of the bones which is the posterior surface. For the ulna we placed the plate under the extensor carpi ulnaris muscle avoiding the subcutaneous border (Figure 2). The AO small fragment set of instruments and implants was used in all the fractures of all the forearms.

This single incision was used in 116 forearm fractures of the shafts of both the radius and ulna in 115 patients (one case had bilateral forearm fractures) in the period from 1994 to 2012. The average duration of the whole procedure was 72 min (from 55 to 110 min). At follow-up (average 5.2 years), four cases were lost leaving 112 forearms in 111 cases. There were no postoperative radioulnar synostosis or neurovascular injuries in any of the forearms. Nonunion occurred in three cases (2.7%).

## Discussion

The three single approaches have limitations and disadvantages. The Boyd and the Colton and Hall approaches carry a high risk for radioulnar synostosis. Bauer et al. [4] reported five cases with synostosis after operating on 12 cases with fractures of both the radius and ulna using the Boyd approach. The biplanar approach of Shenoy needs careful adjustment of his curvilinear incision to the level of the fractures and if the incision was too lateral or too medial exposure of the other bone becomes difficult. This approach was difficult for fractures of the proximal fourth and distal fourth of the forearm shafts. The curvilinear incision leaves a cosmetically less acceptable scar than the straight one.

Most authors prefer two separate incisions than one single incision for exposing both the radius and ulna to avoid the risk of nerve injuries and radioulnar synostosis. However, the incidence of synostosis in the literature ranged from 1.2% to 9% after plate fixation with two separate incisions [4–7]. Also, most authors recommended the anterior Henry approach for the radius. In this approach detachment of muscles is needed such as the supinator, the pronator teres, the flexor digitorum superficilis, the flexor pollicis longus, and the pronator quadrates muscles according to the level of the fractures [8]. This muscle detachment is considered a precursor for radioulnar synostosis. In our series no radioulnar synostosis occurred in any forearm even in the presence of comminution or operative delay which may be due to avoiding detachment of any muscle and using a separate plane of dissection for each bone. In the anterior approach to the radius there is also a hazard of injuries to the neurovascular structures as the radial vessels and their branches, the superficial radial nerve, the median nerve, and the posterior interosseous nerve because most of the neurovascular structures are in the ventral aspect of the forearm. These neurovascular hazards increase when a second operation is needed as plate revision or removal. Langkamer and Ackroyd reported three median nerve injuries, one posterior interosseous nerve injury, and ten permanent superficial radial nerve damage in 44 patients who underwent plate removal through the Henry’s approach for the radius [9]. In our cases there were no neurovascular injuries because this posterior exposure is relatively away from the main neurovascular structures. Although this technique can expose the whole shafts of both the radius and the ulna, we avoided exposure of the far proximal part of the radial shaft to guard against injury to the posterior interosseous nerve and the far distal part of the radial shaft to guard against irritation of the extensor tendons by the implants. We prefer the anterior approach for both of them.

Although the posterior Thompson’s approach can be used for exposing the radius together with an ulnar approach for the ulna, there is difficulty in inspection of reduction and fixation of both the radius and ulna at the same time which is of value in comminuted fractures. Also, the scar of one incision may be better than that of two incisions.

Although the internervous plane for exposure of the radius was between two muscles innervated by the posterior interosseous nerve for both of them, no effect was observed on the grip power which may be due to avoiding any muscle detachment. The internervous plane for exposure of ulnar shaft is the same for all approaches as our technique while in the anterior approach of the radial shaft it is between the brachioradialis muscle innervated by the radial nerve and the pronator teres muscle and the flexor carpi radialis muscles innervated by the median nerve.

In our series the forearm was rested on a board across the chest. This position allowed easier inspection of reduction and fixation of both the radius and ulna at the same time. In the supine position with a side arm board it is difficult to inspect both the radius and ulna at the same time and exposure of the ulna is difficult and usually the surgeons flex the elbow with the forearm away from the side board.

In conclusion this straight posterior incision with the forearm rested on a board across the chest allowed easy and relatively safe exposure of fractures of the mid shafts of both the radius and ulna.

## Conflict of interest

The author declares that he has no conflict of interest.

## References

[1] Boyd HB (1940) Surgical exposure of the ulna and proximal third of the radius through one incision. Surg Gynecol Obstet 71, 86–88.

[2] Colton CL, Hall AJ (1991) Atlas of orthpaedic surgical approaches. Oxford, Butterworth Heinman, pp. 192–193.

[3] Shenoy RM (1995). Biplanar exposure of radius and ulna through a single incision. J Bone Joint Surg Br 77(4) 568–570. 7615599

[4] Bauer G, Arand M, Mutschler W (1991) Posttraumatic radioulnar synostosis after forearm fracture osteosynthesis. Arch Orthop Trauma Surg 110(3), 142–145. 2059537 10.1007/BF00395796

[5] Anderson LD, Sisk D, Tooms RE, Park WI III (1975) Compression plate fixation in acute diaphyseal fractures of the radius and ulna. J Bone Joint Surg Am 57(3), 287–297. 1091653

[6] Stern PJ, Drury WJ (1983) Complications of plate fixation of forearm fractures. Clin Orthop Relat Res 175, 25–29. 6839596

[7] Vince KG, Miller JE (1987) Cross-union complicating fracture of the forearm. Part 1: adults. J Bone Joint Surg Am 69(5) 640–653. 3110165

[8] Henry AK (1957) Extensile exposure. Baltimore, Williams-Wilkins, pp. 100–107.

[9] Langkamer VG, Ackroyd CE (1990) Removal of forearm plates. A review of the complications. J Bone Joint Surg Br 72(4) 601–604. 2380210 10.1302/0301-620X.72B4.2380210

